# A Rare Case of Leptomeningeal Carcinomatosis Secondary to Metastatic Non-Small Cell Lung Carcinoma

**DOI:** 10.7759/cureus.25436

**Published:** 2022-05-28

**Authors:** Salina Munankami, Manish Shrestha, Sijan Basnet, Swarup Sharma Rijal

**Affiliations:** 1 General Medicine, Kathmandu Medical College, Kathmandu, NPL; 2 Internal Medicine, Tower Health Medical Group, Reading, USA; 3 Internal Medicine, Tower Health Medical Group, Wyomissing, USA

**Keywords:** cerebrospinal fluid (csf), brain metastasis, intrathecal chemotherapy, non-small cell lung carcinoma, leptomeningeal carcinomatosis

## Abstract

Leptomeningeal carcinomatosis is a rare complication of metastatic systemic malignancy, with lung cancer being the most common cause. We present a case of a 75-year-old man with a past medical history of right non-small cell lung carcinoma and ischemic stroke who presented with a persistent headache and swallowing difficulties. On evaluation, the patient was initially diagnosed with a subacute infarct of the right posterior frontal lobe following magnetic resonance imaging (MRI). The patient’s headache and dysphagia worsened, increasing the possibility of brain metastasis. The patient underwent cerebrospinal fluid analysis including cytology and multiple MRI studies with no obvious explanation for the symptoms. The patient eventually developed multiple cranial nerve palsies, and a diagnosis of leptomeningeal carcinomatosis was made with neuroradiology consultation for the MRI.

## Introduction

Leptomeningeal carcinomatosis (LMC) is an infrequent complication of malignancy that results following the dissemination of the tumor cells into the subarachnoid space. Lung cancer is the most common systemic malignancy for metastasis followed by gastric cancer, breast cancer, malignant lymphoma, and malignant melanoma [1]. Around 10%-26% of patients with lung cancer ultimately develop leptomeningeal metastasis [2]. As a result of frequent multifocality, clinical signs and symptoms depending on the site of involvement are often non-specific. However, typical findings include cranial nerve palsy, raised intracranial pressure, or meningeal irritation leading to diplopia and facial weakness, changes in hearing, and headache, respectively [3]. Magnetic resonance imaging (MRI) of the brain and cerebrospinal fluid (CSF) analysis are the standard for diagnosis of the condition [4,5]. However, Straathof et al. [6] concluded that in the absence of a gold standard test for diagnosis, CSF cytology had a sensitivity and specificity for diagnosing the condition to be 75% and 100%, respectively, whereas with gadolinium MRI, the sensitivity and specificity for diagnosing the condition were 76% and 77%, respectively. The sensitivity of enhanced MRI was equivalent to that of CSF analysis and the specificity of CSF examination was higher than that of enhanced MRI, being 100% and 77%, respectively [6]. The patient developed leptomeningeal metastasis in the present case, which was not demonstrated following a series of multiple MRIs and CSF cytology.

## Case presentation

A 75-year-old man with a past medical history of stage IIIA adenocarcinoma of the right lung (treated with chemotherapy/resection in 2009 and radiotherapy for local recurrence in 2019), adenocarcinoma of the rectum (treated with resection and colo-colonic anastomosis in 2009), and lacunar infarct of the left thalamus (March 2017) presented on April 13, 2020, with persistent right-sided headache and difficulty swallowing for two to three months. The patient described headache as a constant pressure-like sensation localizing to the right parietotemporal region that was somewhat relieved with ibuprofen. There was no history of falls or injuries, jaw claudication, or weakness in the shoulders or legs. He had an extensive outpatient workup and was seen by an ophthalmologist, an otolaryngologist, and a dentist without any identifiable cause except for suspected temporal mandibular dysfunction that was unresponsive to treatment with nonsteroidal anti-inflammatory drugs and physical therapy. MRI of the head with and without contrast performed a week ago for similar complaints showed a small 4-mm rounded focus of bright signal on diffusion-weighted imaging with surrounding peripheral cortical enhancement, a probable small subacute right posterior frontal lobe cortical infarct (arterial or venous in a setting of cortical vein thrombosis) rather than treated metastasis. The patient was started on aspirin and atorvastatin and referred to neurology outpatient with a plan for a repeat MRI of the head in four weeks. The patient also had difficulty swallowing both solids and liquids with occasional coughing episodes for a month. He had lost about 7 lbs in a month. The patient had multiple admissions for dysphagia with poor appetite and underwent extensive workup by gastroenterology, neurology, and otolaryngology without a mechanical explanation. On physical examinations, the patient did not have significant deficits of cranial nerves (CNs) I-XI, no focal neurologic abnormalities, and preserved strength and sensation in the bilateral face and upper and lower extremities. The patient was admitted with a diagnosis of subacute stroke of the right posterior frontal lobe cortical infarct. The patient underwent computed tomography angiography of the head and neck, which did not show any hemodynamically significant stenosis. A transthoracic echocardiogram showed no obvious regional wall abnormalities with a left ventricular ejection fraction of 56%, evidence of probable patent foramen ovale (PFO) with a right to left shunting across interatrial septum on agitated saline injection, mild mitral regurgitation, and tricuspid regurgitation. The cardiology team recommended managing the PFO conservatively. The stroke was determined to be cryptogenic. The cardiology team recommended a 30-day event monitor with consideration for a loop recorder, a repeat MRI with contrast in four weeks, aspirin, and atorvastatin, and a follow-up with outpatient vascular neurology. The patient was evaluated by a speech and language pathologist with suspicion of the cause of mild oropharyngeal dysphagia that was related to subacute stroke and appeared to be at low to moderate risk for postprandial aspiration. The patient was discharged on April 15, 2020, on gabapentin for headaches and prednisolone for concern with giant cell arthritis, and was advised to follow up with ophthalmology as an outpatient. The patient was readmitted on April 24, 2020, due to difficulty in swallowing, regurgitation, and inability to swallow his medications. Otolaryngology performed a laryngoscopy, which did not reveal any pathology with no nasal/laryngeal/pharyngeal mass, normal vocal fold movement, and mild inter-arytenoid edema and dysphagia possibly related to right frontal cerebrovascular accident. Fluoroscopic swallowing function with video showed no evidence of cervical esophageal mass, web, or diverticulum. The patient tolerated pureed feeds and hence was discharged again on April 27, 2020.

The patient was readmitted on May 5, 2020, with complaints of worsening headache in the right frontoparietal region, occasional blurry vision, perioral numbness, and worsening dysphagia. On physical examination, the patient had right upper eyelid ptosis. The patient had mild right-sided facial droop, flattening of the right nasolabial fold, and inability to close the right eye completely, which were suggestive of CNs III, V, and VII palsy. He had preserved strength and sensation in bilateral upper and lower extremities. Basic metabolic profile and complete blood count with differential count were unremarkable. Thyroid-stimulating hormone (TSH) levels were 1.51. Voltage-gated calcium channel antibody, anti-muscle specific kinase (anti-MuSK) antibodies, LRP4 (low-density lipoprotein receptor protein) autoantibody, paraneoplastic panel, angiotensinogen converting enzyme (ACE) levels, Lyme antibody, SSA(Ro)/SSB(La) autoantibodies, creatine kinase, creatine phosphokinase, anti-lupus antibody, lupus anticoagulant, anti-double-stranded DNA antibodies, anti-neutrophil cytoplasmic antibody (ANCA), lupus anticoagulant antibody, anti-centromere Ab, rheumatoid factor, human immunodeficiency virus (HIV), and ganglioside antibody were negative. MRI of the head with and without contrast showed diffusion hyperintense right frontal lobe lesion and associated findings that were not significantly changed from April 09, 2020. The stability for nearly a month increased the odds that this represented metastasis. A magnetic resonance venogram performed for suspected venous sinus thrombosis was negative. MRI of the cervical spine was negative for cord signal abnormality, pathological enhancement, or osseous metastatic disease. The patient underwent a lumbar puncture, which was negative for cytology, cryptococcal antigen, meningitis-encephalitis panel by polymerase chain reaction (PCR), flow cytometry, Venereal Disease Research Laboratory (VDRL), enterovirus PCR, herpesvirus 6 PCR, varicella-zoster PCR, herpes simplex virus PCR, cytomegalovirus PCR, West Nile virus PCR, and acid-fast bacilli stain and culture. Temporal artery biopsy was negative for giant cell arteritis. The patient was started on carbamazepine for suspected trigeminal neuralgia. The patient's neurologic deficits continued to worsen with decreased oral intake and malnutrition. The patient was discharged home on May 15, 2020, with home health care.

**Figure 1:**
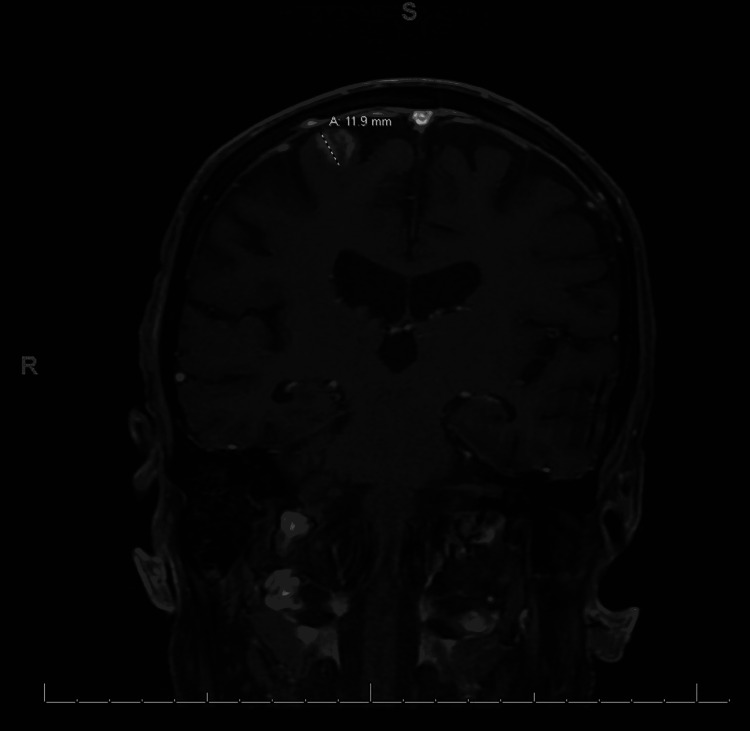
Image showing stable right frontal lobe lesion, which was unchanged from previous MRIs a month ago, likely metastasis.

**Figure 2:**
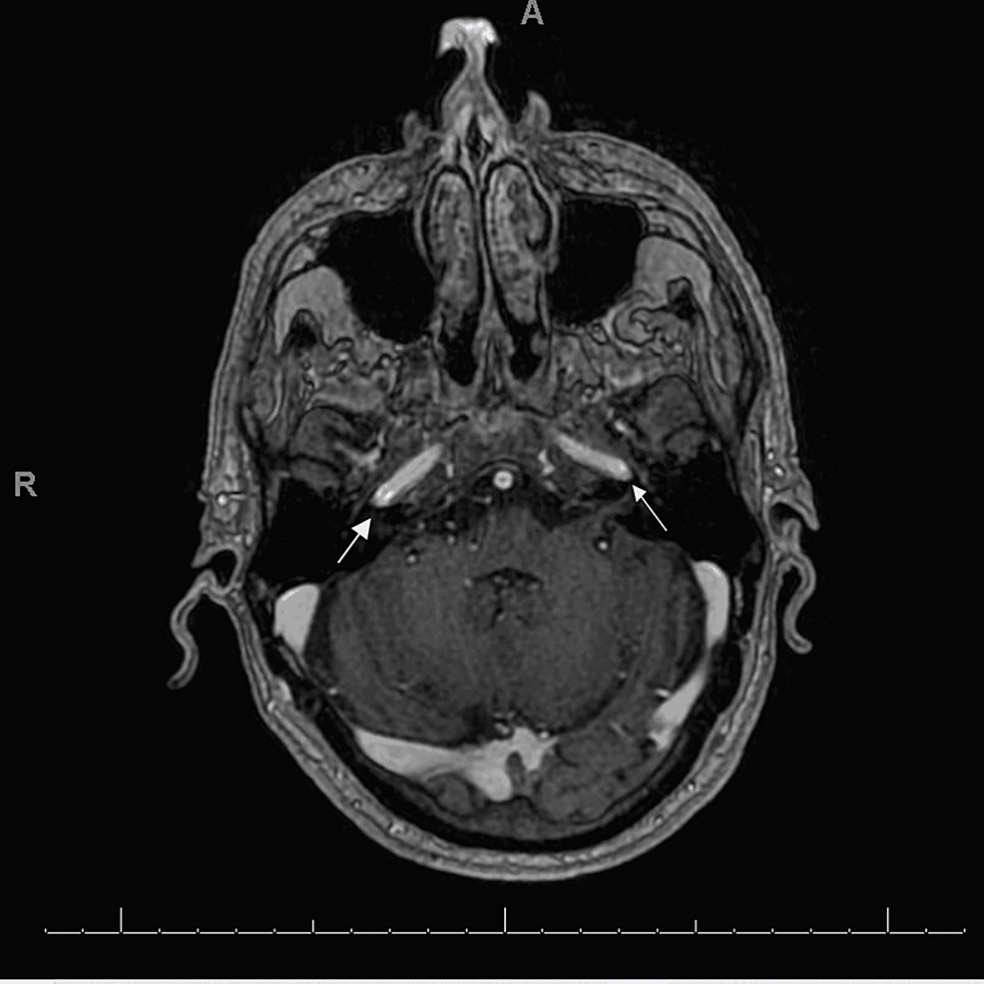
Image showing symmetric seventh and eighth nerve enhancement and some slightly nodular enhancement of the right trigeminal nerve suggestive of leptomeningeal metastases.

The patient was readmitted once more with failure to thrive secondary to persistent dysphagia on May 18, 2020. The patient had persistent CNs III, V, and VII palsy. A repeat MRI was performed for worsening dysphagia, persistent headache, and multiple CN palsy without notable cause. MRI of the head with and without contrast showed a 12-mm enhancing focus superiorly in the right precentral cortex with associated FLAIR hyperintensity (Figure 1) and a tiny focus of high diffusion signal similar to that found on May 6, 2020, a 9-mm enhancing focus inferiorly in the right cerebellar hemisphere that was difficult to perceive on previous MRI due to posterior fossa artifacts but in retrospect was probably present on both examinations, and a focal FLAIR hyperintensity superior medial in the left frontal lobe without enhancement that was probably a small area of gliosis. Neuroradiology consultation for the MRI showed symmetric seventh/eighth nerve enhancement with some slightly nodular enhancement of trigeminal nerve findings suggestive of leptomeningeal metastasis (Figure 2). The patient was started on steroids and was planned for palliative whole-brain radiation as an outpatient and discharged home. The patient passed away shortly after at his home.

## Discussion

LMC or carcinomatous meningitis is the infiltration of leptomeninges by the malignant cells, which is a devastating metastatic complication of solid tumors or hematological malignancies with high mortality and dismal prognosis [7]. The overall incidence of LMC for all types of systemic cancer was documented to be 3-8%, but in an autopsy series, it was noted to be up to 20%, with lung malignancy being the most common systemic malignancy for the disease [1,8].

LMC often presents as multifocal neurologic deficits because of infiltration of cranial and spinal nerve roots, direct invasion of the brain or spinal cord, obstructive hydrocephalus, or a combination of these factors. As a result, the patient may suffer from headache, nausea/vomiting, change in mental status, diplopia, facial numbness/palsy, hearing loss, loss of visual equity, paresthesia, pain in the back or neck, weakness in the legs, and bowel/bladder dysfunction [9]. In our case, the patient presented with persistent headaches with progressive involvement of the facial nerve and trigeminal nerve causing facial palsy and facial numbness, respectively. A detailed clinical history is critical to differentiate from other diagnoses that can be present with identical manifestations such as infectious meningitis, metabolic and toxic encephalopathies, sarcoidosis, paraneoplastic syndromes, and chemoradiation side effects. Heightened clinical understanding of LMC allows for earlier detection and treatment, maintenance of the quality of life, and prolonging survival [9].

The diagnosis of LMC is accomplished with gadolinium-enhanced MRI and CSF cytology. An MRI of the brain with whole-spine imaging with T1- and T2-weighted sequences with contrast is recommended if LMC is suspected of metastatic malignancies that can involve the entire central nervous system (CNS) [9]. However, the sensitivity of MRI has been reported to be between 65% and 75% only [10]. The gold standard for diagnosis is the presence of malignant cells in CSF. However, false-negative results are up to 50% in observational studies [11]. Hence, serial CSF sampling can improve sensitivity [12]. MRI findings are generally only abnormal in 75-90% of the patients with cytology-positive CSF. Therefore, neither MRI nor CSF sampling is sensitive enough when used alone for the diagnosis of LMC. Eventually, clinical along with MRI findings or serial CSF analysis should be performed for the diagnosis of LMC [13].

LMC usually has a prognosis of three to four months [14]. An evolving number of systemic anticancer therapies especially molecular targeted drugs and immunotherapies that cross the blood-brain barrier need to be individualized based on patient characteristics. In patients with non-small cell lung carcinoma (NSCLC), systemic administration of genotype-directed target therapies can result in clinical benefits. Epidermal growth factor receptor (EGFR) tyrosine kinase inhibitors such as erlotinib and osimertinib are preferred in EGFR-mutant NSCLC [15,16]. Anaplastic lymphoma kinase fusion oncogene-positive NSCLC can be treated with ALK inhibitors such as loratinib, which has increased CNS penetration and intracranial activity [17]. Intrathecal chemotherapy has historically been a primary treatment for LMC in patients with solid tumors, although its efficacy is modest, and its superiority compared with systemic treatment has not been well established in randomized studies [18]. Bulky symptomatic disease sites can be treated with radiation therapy with whole-brain radiation for diffuse encephalitis or hydrocephalus. Persistent hydrocephalus can be treated with steroids or ventricular peritoneal shunt. Identifying patients with a quite poor prognosis can help limit unnecessary or futile interventions and maximize supportive care and comfort [18,19].

## Conclusions

LMC is a rare complication of NSCLC with variable sensitivity of CSF sampling and MRI, both being the standard for diagnosis of the disease. Patients with negative MRI and CSF cytology might need serial MRIs with neuroradiology consultation and CSF cytology if the suspicion of the disease remains high as there is a high prevalence of false-negative results even when both are performed together for the diagnosis of LMC.
